# Implementation of AD BBMs: Approach in Memory Clinics in the Netherlands

**DOI:** 10.1002/alz70857_099978

**Published:** 2025-12-24

**Authors:** Floor Duits, Paula I. Vanneste, Niki S.M. Schoonenboom, Nicolaas A. Verwey, Salka S Staekenborg, Astrid M van Strien, Hugo Aben, Argonde C. van Harten, Charlotte E. Teunissen

**Affiliations:** ^1^ Alzheimer Center Amsterdam, Department of Neurology, Vrije Universiteit Amsterdam, Amsterdam UMC location VUmc, Amsterdam, Netherlands; ^2^ Amsterdam Neuroscience, Neurodegeneration, Amsterdam, Netherlands; ^3^ Alzheimer Center Amsterdam, Neurology, Vrije Universiteit Amsterdam, Amsterdam UMC location VUmc, Amsterdam, Netherlands; ^4^ Department of Neurology, Spaarne Gasthuis location Haarlem, Haarlem, the Netherlands, Haarlem, Netherlands; ^5^ Medisch Centrum Leeuwarden, Department of Neurology, Leeuwarden, Netherlands; ^6^ Medisch Centrum Tergooi, Department of Neurology, Hilversum, Netherlands; ^7^ Department of Geriatric medicine, Jeroen Bosch Ziekenhuis, Den Bosch, Netherlands; ^8^ Elizabeth Tweesteden Ziekenhuis, Department of Neurology, Tilburg, Netherlands; ^9^ Alzheimer Center Amsterdam, Department of Neurology, Amsterdam UMC, location VUmc, Amsterdam, Netherlands; ^10^ Amsterdam Neuroscience, Neurodegeneration, Amsterdam, Noord‐Holland, Netherlands; ^11^ Neurochemistry Laboratory, Department of Laboratory Medicine, Amsterdam Neuroscience, Amsterdam UMC, Vrije Universiteit Amsterdam, Amsterdam, Netherlands

## Abstract

**Background:**

In current clinical practice, a diagnosis of Alzheimer's disease (AD) is often delayed and inaccurate. Blood‐based biomarkers (BBMs) are emerging as promising tools for accurate detection of AD. However, there is a need for real‐world evidence to bridge the gap between findings in research cohorts and impact on clinical practice. Therefore, we aim to study clinical impact and diagnostic utility of BBMs for AD in memory clinics as part of the Davos Alzheimer's Collaborative System Preparedness (DAC‐SP) Accurate Diagnosis project.

**Method:**

We initiated project planning discussions with clinicians of regional memory clinics in the Netherlands. Clinicians (neurologists/geriatricians) saw BBMs as a potential improvement of diagnostic possibilities and were keen to gain experience with BBMs. However, few clinics had sufficient infrastructure to conduct trials and time restraints were a limiting factor. Therefore, we designed two studies. First, a diagnostic randomized controlled trial with six‐months follow‐up will randomize participants (*n* = 550) after venepuncture for disclosure versus no disclosure of BBM results (ptau‐217 and serum NfL). Outcome measures are change in patient management, diagnosis, diagnostic certainty and time to final diagnosis. The second study is a pragmatic study of the impact of BBMs on clinicians’ decision‐making when they are used in routine practice (intended *n* >450). In both studies, patients >55 years old can participate when they present with cognitive complaints in a memory clinic in the Netherlands.

**Result:**

IRB approval for the studies is pending. Expected start of recruitment is Q1 of 2025. Currently, CSF or PET biomarkers are not routinely performed in memory clinics (range 0‐25% of cases). Therefore, we hypothesize BBMs can improve diagnostic accuracy and confidence in the diagnosis and shorten time to diagnosis. In the session, emerging learnings will be shared on implementation and impact of BBMs on the diagnostic process.

**Conclusion:**

AD BBMs are approaching clinical practice at a rapid pace and may have substantial impact on clinical practice. Memory clinic physicians see their potential advantages. These studies will be useful to familiarize them with the use of these biomarkers, and develop appropriate use criteria for BBMs in the daily practice of Dutch memory clinics.

